# Book Review

**Published:** 1899-03

**Authors:** 


**From Our Dead Selves to Higher Things.** By FREDERICK JAMES GANT. Second Edition. Pp. xiv., 185. London: Baillière, Tindall & Cox. 1897.—It is always refreshing to find that a busy surgeon has time to write on matters other than those concerning his profession, and most of us will be the better for reading this expression of the author's inner thoughts. The high-toned morality of the work is unquestionable, but to say with the author that "the veil of 'Agnosticism' is rent in twain from the top to the bottom" depends on the point of view of the reader.

**The Determination of Sex.** By Dr. LEOPOLD SCHENK. Pp. 173. London: The Werner Company. 1898.—The first half of this book is a discursive account of the various theories which have been held as to the determination of the sex of children. The latter half deals with Dr. Schenk's researches on the subject. They amount to this—that many women, though not considered to be diabetic, pass minute traces of sugar in the urine. Such women generally have female offspring. If these traces of sugar be removed by a diet consisting mainly of proteids and fats, males are produced. The evidence offered in support of this theory is most meagre and unsatisfactory. Some things doubtless are better done in Germany; but it is refreshing, after looking over this volume, to turn to the *Evolution of Sex*, by Geddes and Thomson, who give a lucid account of what is known on the subject.

**Rheumatoid Arthritis.** By GILBERT A. BANNATYNE, M.D. Second Edition. Pp. xii., 182. Bristol: J. Wright & Co. 1898.—There has been some revision and improvement in this book, which now forms a fairly complete work on the subject. The theory advanced in the first edition as to a microbic origin for the disease is altered in some respects, the author coming to the conclusion that there are at least two forms of rheumatoid arthritis—the one acute and undoubtedly of microbic character, the other chronic and degenerative. No evidence, however, is given as to whether the micro-organisms characteristic of the acute forms are found in the chronic ones, and the relationship of the two is left indefinite. The treatment advocated is much the same as in the former edition. A regrettable point is that the author barely mentions the excellent results which have been obtained by the action of high temperatures, by the hot air or electric radiant baths.

**The Extra Pharmacopœia.** By WILLIAM MARTINDALE and W. WYNN WESTCOTT, M.B. Ninth Edition. Pp. xxviii., 626. London: H. K. Lewis. 1898.—A work which in a comparatively few years has reached its ninth edition stands in need of no commendatory criticism. The British Pharmacopœia becomes continuously more and more inadequate to fulfil the requirements of practice, and a text-book of information such as this present excellent work is now a necessity. In this last edition have been incorporated all the essential contents of the new B.P., while in addition there is a wondrous store of instruction about the multitude of remedial agents in the use of which the B.P. in its narrow scope does not take notice. A monument of industry, of up-to-date learning, and of usefulness, this book is indispensable to every intelligent practitioner who desires to employ the full therapeutical resources of his art.

**Chavasse's Advice to a Wife.** Revised by FANCOURT BARNES, M.D. Fourteenth Edition. Pp. 306. London: J. & A. Churchill. [1898.]—We consider that this edition maintains the reputation that previous ones have made for this very useful work. The introductory chapter on the health of the young wife is very good, and can be read with advantage by all women. The portion that follows is more or less pertinent to the subject of pregnancy, and can be recommended as a very good guide.

**Chavasse's Advice to a Mother.** By GEORGE CARPENTER, M.D. Fifteenth Edition. Pp. x., 430. London: J. & A. Churchill. [1898.]—Few books of this character, we believe, have proved more popular and useful than this, and we are bound to say that it is head and shoulders above the usual manuals intended to instruct mothers on their duties towards their babies. In revising the present edition and relegating the few prescriptions to an appendix, Dr. Carpenter has done well and wisely. About such light and airy conversations as we find here there is a charm which has always attracted us ever since we learnt our first lessons of science from Dr. Brewer's *Guide to Knowledge;* but no book of this kind that we have ever seen has treated us to so many quotations from the poets: Byron, Coleridge, Martin Tupper (!), Shakspere, Barham, Knox, Tennyson, and many others are quoted; while even the author, under the influence of the question of the propriety of sucking one's thumb, breaks out into verse. We commend the advice to the mother on her behaviour to her doctor on pp. 323–5, and only wish we were always treated with so much consideration.

**Brief Essays on Orthopædic Surgery.** By NEWTON M. SHAFFER, M.D. Pp. v., 81. New York: D. Appleton and Company. 1898.—Those interested in orthopædic surgery as a speciality will do well to read these short essays, which are reprinted from various periodicals. They define the position of the orthopædic surgeon and urge the desirability of conservatism. They deplore the tendency of some specialists—gynæcologists, for example—to commit depredations in the fields of general surgery.

**The Diseases of Children's Teeth, their Prevention and Treatment.** By R. DENISON PEDLEY. Pp. xi., 268. London: J. P. Segg & Co. [1895.]—Considerable praise is due to the author for his attempt to interest medical students and practitioners in this subject. The book is very readable, not too long, and contains very practical hints. The chapters on oral hygiene and irregularities of position are excellent. It is a pity that the author advocates a proprietary tooth powder. Still, on the whole his book fulfils its aim as well as any work of the kind with which we are acquainted.

**Diseases of the Upper Respiratory Tract.** By P. WATSON WILLIAMS, M.D. Third Edition. Pp. xvi., 282. Bristol: John Wright & Co. 1898.—This work has evidently met a well-marked requirement. It contains in a small compass much valuable information, and though it is practically a reprint of the previous issue, it is well up to date and forms a handy guide to the subject.

**The Johns Hopkins Hospital Reports.** Vol. VII. Nos. 1–2 — *Report in Gynecology*. Baltimore: The Johns Hopkins Press. 1898.—The greater part of this report is taken up with a "critical review of seventeen hundred cases of abdominal section from the standpoint of intraperitoneal drainage," wherein the observations of the author, Dr. J. G. Clark, go to show that the drainage-tube is still used too largely in abdominal work. The rest of the book is concerned with "the etiology and structure of true vaginal cysts," written by Dr. J. E. Stokes. Of the merit of these reports we have frequently spoken, and the present work further enhances the solid reputation which they have gained for accurate and painstaking research.

**The Middlesex Hospital. Reports of the Medical, Surgical, and Pathological Registrars for the Year 1896**. London: H. K. Lewis. 1897.—These reports comprise an elaborate classification of all the cases in the hospital during the year, with abstracts of the more important ones and of *post-mortem* examinations. Attention may be directed to the tables on diphtheria, the classification of cases of hernia, and of over thirty cases of operation on the kidneys. Seven out of sixteen cases of strangulated hernia were fatal, a high mortality now-a-days. Thirty-one cases of appendicitis were admitted to the medical wards, twenty of which recovered, one died, and the rest were transferred to the surgical wards, where twenty-three cases were admitted altogether, five being fatal. In thirty operations on the kidney, two only were followed by death. It is interesting to note that seven of these proved negative explorations, four for nephralgia and three for hæmaturia, *i.e.* nearly 25 per cent.

**Clinical Report of the Rotunda Hospitals, 1896–97.** Dublin: John Falconer. 1898.—The master and his assistants are highly to be congratulated both on the excellent results obtained, and the manner in which the results are tabulated and presented for perusal. Three thousand four hundred and fifty-five cases were attended, with a total maternal mortality of seven or ·205 per cent. Only two deaths are attributed to sepsis. The number of cases in which the temperature reached 100.8° F. was seventy-eight, but in only fifteen was it considered necessary to wash out the uterus. A review of the work of the gynæcological department contains a very interesting account of the arrangements of the operating theatre, methods of sterilising hands, clothing, instruments, sutures, ligatures, etc. In all plastic operations on the vagina, a preliminary curetting of the uterus is done: this procedure might be more widely adopted with advantage. The lists of operations, the mortality of the same, and the reasons explaining the deaths should all be read in the original paper. The mortality of eleven vaginal cœliotomies, and fifty-five ventral cœliotomies, including nine panhysterectomies, was eight or twelve per cent.

**Transactions of the College of Physicians of Philadelphia.** Vol. XIX. Philadelphia: Printed for the College. 1897.—The usual obituary notices and presidential address are followed by a selection from the papers read during the year. One of the most noteworthy of these is by Dr. Alfred Stengel on the value of auscultatory percussion in diagnosis. The different methods of stethoscopic percussion are described, and the author gains confidence in the method in accordance with increasing experience: his general conclusions are in accord with other writers, who have learnt the method in one or other of its various forms. A paper by Dr. Charles Winslow Dulles advocates the view "that consumption is not a disease that is now spreading," but that "for many years there has been a comparatively steady decline in the proportion of cases of consumption": he combats the current views in favour of contagion, and entertains the hope that without spasmodic and violent measures the disease will soon cease to be the scourge and terror that it now is.

**Transactions of the American Pediatric Society.** Vol. IX. Reprinted from *The Archives of Pediatrics.* 1897.—Several interesting papers will be found in these records; notably a paper by Dr. J. O'Dwyer on retained intubation tubes, based on an experience of over five hundred cases of intubation. Dr. Koplik describes his method of rapid bacteriological diagnosis of diphtheria in two to three hours. There is an interesting paper on several cases of retro-œsophageal (intrathoracic) abscess presenting anomalous symptoms, and on five cases of cerebral abscess in infants. Empyema, pneumonia, and many other interesting subjects are considered.

**Transactions of the Dermatological Society of Great Britain and Ireland.** Vol. III. London: H. K. Lewis. 1897.—There seems to be life in this Society, for the volume before us contains more than double the matter of the previous one. The address by the President, Dr. J. F. Payne, on "Bacteria in Diseases of the Skin," is well worth perusal; it contains many deep thoughts, and there is no doubt that the "pathology of this organ [skin] is decidedly in advance of that of many other parts in regard to the subject of inflammation." The article on "Yaws in India" by Mr. Arthur Powell is almost exhaustive of the subject; and "The Influence of Light on the Skin," by Dr. R. L. Bowles, shows the competency of the author to discourse on the subject. We should like more pictures of the quality of that which illustrates Dr. Abraham's case of pustular eruption from tar..

**The Year-Book of Treatment for 1899.** London: Cassell and Company, Limited.—This, the fifteenth annual issue, is excellent in point of merit and general usefulness to the practitioner, and in saying this, we afford it the best recommendation to all those who are not acquainted with this summary of the year's progress. We note one new feature this year in the able article by Dr. Burton-Fanning on "The Open-air Treatment of Phthisis."

**Index-Catalogue of the Library of the Surgeon-General's Office, United States Army.**—Second Series. Vol. III., C—Czygan. Washington: Government Printing Office. 1898.—The present volume of this monumental work bears the same impress of elaborate care and indefatigable energy which has characterised the previous volumes. It is indispensable to all libraries and writers on medical subjects.

**The Journal of Tropical Medicine.** London: John Bale, Sons & Danielsson, Ltd.—This is a monthly Journal devoted to medical, surgical, and gynæcological work in the Tropics, and is under the direction of Mr. James Cantlie and Dr. W. J. Simpson, whose long experience of disease in the East entitles them to speak with authority on the subject of tropical medicine. It began life in August, 1898, and has been added to our exchange-list. Amongst noteworthy contributions to the earlier numbers of this periodical are articles—by Sir Joseph Fayrer, "Enteric Fever among British Soldiers in India"; by Haffkine and Bannerman, "The Testing of Haffkine's Plague-Prophylactic in Plague-Stricken Communities in India"; by Rogers on "The

Distribution and Harmfulness of the Anchylostomum"; and by Voorthuis on "Unna's New Method of Treating Leprosy." Space is also devoted to an epitome of recent literature on tropical medicine and to reviews of books bearing upon this subject.

**Journal of the Boston Society of Medical Sciences.** Boston, U.S.A.—We welcome this Journal as a new exchange. It contains papers of more than local interest, and shows that the Boston Society is doing very good work. With the Medical School of Harvard University and the various other scientific institutions in Massachusetts, its opportunities are great. The opening number of the new volume contains a careful investigation into "The minute Anatomy of the Oblongata and Pons of the Chimpanzee, with special Reference to their Homologies with Man."

---

